# Parabiosis reveals the correlation between the recruitment of circulating antigen presenting cells to the retina and the induction of spontaneous autoimmune uveoretinitis

**DOI:** 10.1186/s12974-022-02660-2

**Published:** 2022-12-09

**Authors:** Scott W. McPherson, Neal D. Heuss, Md. Abedin, Heidi Roehrich, Mark J. Pierson, Dale S. Gregerson

**Affiliations:** Department of Ophthalmology and Visual Neurosciences, University of Minnesota, 2001 6th Street SE, Lions Research Building, Room 482A, Minneapolis, MN 55455 USA

**Keywords:** Retina, Microglia, Antigen presenting cells, Autoimmunity, Origin, Parabiosis

## Abstract

**Background:**

Characterizing immune cells and conditions that govern their recruitment and function in autoimmune diseases of the nervous system or in neurodegenerative processes is an area of active investigation. We sought to analyze the origin of antigen presenting cells associated with the induction of retinal autoimmunity using a system that relies on spontaneous autoimmunity, thus avoiding uncertainties associated with immunization with adjuvants at remotes sites or adoptive transfer of in vitro activated T cells.

**Methods:**

R161H mice (B10.RIII background), which spontaneously and rapidly develop severe spontaneous autoimmune uveoretinitis (SAU), were crossed to CD11c^DTR/GFP^ mice (B6/J) allowing us to track the recruitment to and/or expansion within the retina of activated, antigen presenting cells (GFP^hi^ cells) in R161H^+/−^ × CD11c^DTR/GFP^ F_1_ mice relative to the course of SAU. Parabiosis between R161H^+/−^ × CD11c^DTR/GFP^ F_1_ mice and B10.RIII × B6/J F_1_ (wild-type recipient) mice was done to explore the origin and phenotype of antigen presenting cells crucial for the induction of autoimmunity. Analysis was done by retinal imaging, flow cytometry, and histology.

**Results:**

Onset of SAU in R161H^+/−^ × CD11c^DTR/GFP^ F_1_ mice was delayed relative to B10.RIII-R161H^+/−^ mice revealing a disease prophase prior to frank autoimmunity that was characterized by expansion of GFP^hi^ cells within the retina prior to any clinical or histological evidence of autoimmunity. Parabiosis between mice carrying the R161H and CD11c^DTR/GFP^ transgenes and transgene negative recipients showed that recruitment of circulating GFP^hi^ cells into retinas was highly correlative with the occurrence of SAU.

**Conclusions:**

Our results here contrast with our previous findings showing that retinal antigen presenting cells expanding in response to either sterile mechanical injury or neurodegeneration were derived from myeloid cells within the retina or optic nerve, thus highlighting a unique facet of retinal autoimmunity.

## Background

Understanding the conditions that promote or suppress the recruitment of lymphocytes and myeloid cells into central nervous system (CNS) tissue and whether it results in an effector or a protective/regulatory immune response is an active area of investigation with practical consequences for treating inflammatory diseases and neurodegenerative conditions of the CNS. The vast majority of studies concerning immune cell recruitment to the CNS have concentrated on the brain and spinal cord. However, determining the origin, activation/recruitment conditions, and functional output of immune cells, particularly myeloid cells, within or into these tissues can be problematic due to a substantial presence of circulation-derived macrophages in the perivascular spaces and meninges of the brain and spinal cord that are distinct from resident myeloid cells (microglia, MG) in the parenchyma [1].

The retina is uniquely suited to study CNS myeloid cell maintenance, replacement, and function in that it can be easily removed and cleaned of adjacent tissue, lacks meninges, and is thus a truer representation of neural parenchyma. We have found that recruitment of new myeloid cells to the retina is highly dependent on the stimulus. Using CD11c^DTR/GFP^ reporter mice [2], we found an expansion of GFP^hi^ myeloid cells (GFP^hi^ MG) within the retina in response to cone photoreceptor cell degeneration [3] as well as to the limited injury of retinal cells induced by either optic nerve crush, intense light exposure, or partial optic nerve transection [4, 5]. Subsequent fate mapping, depletion, and parabiosis experiments revealed that the nascent GFP^hi^ cells found after these injuries were not derived from circulating progenitors but rather were derived from resident MG in either the retina or the optic nerve [4]. In contrast, we found that ablation of resident myeloid cells and MG from the retina by radiation followed by CD11c^DTR/GFP^ bone marrow transplant revealed a slow replacement by circulating donor myeloid cells that were primarily GFP^lo^ [6]. Furthermore, optic nerve crush injury to these radiation bone marrow chimeras greatly stimulated the replacement of host with donor myeloid cells within the retina and the development of GFP^hi^ cells from the donor myeloid precursors [6].

Studies from other laboratories also support the idea that myeloid cell recruitment to the retina is dependent on the stimulus. While the renewal rate of MG in the quiescent retina is unknown, it was initially speculated that they were continually replaced by circulating myeloid progenitors [7, 8]. However, these studies were done with irradiated bone marrow chimeric mice. It was subsequently demonstrated that under normal, healthy conditions the entry of circulating monocytes into the CNS is highly limited [9–11] and that retinal MG in particular are sustained as a closed, self-renewing population [12, 13]. Studies involving genetic and chemical depletion of retinal MG showed that replacement MG were either derived solely from residual retinal MG [14] or from MG in the optic nerve and ciliary body/iris [15]. Conversely, in certain severe retinal injury models, newly recruited retinal MG were shown to be derived from circulating monocytes [12, 16–18]. Regardless of their origin, the retinal microenvironment compels newly arrived myeloid cells to be the functional equivalent of the endogenous MG [6, 12], although those derived from circulating monocytes do retain some distinguishing phenotypic signatures [13].

Although retinal autoimmunity is a fundamentally different process than either the injury or neurodegeneration responses discussed above, determining the origin and role of both the endogenous MG as well as recruited myeloid cells will be crucial to understanding the immunopathogenesis of neuroinflammation. Early studies demonstrated the critical role of myeloid cells in both the induction and resolution of experimental autoimmune uveoretinitis (EAU), a rodent model of retinal autoimmunity [19–21], but could not distinguish the function of resident MG and infiltrating monocytes. More recent studies with immunization-induced EAU suggested that resident MG are crucial for the retinal infiltration of immune cells from the circulation [22] and that their population expands along the course of the disease [23]. However, these studies were limited by several factors. First is the reliance on adoptive transfer of activated T cells or self-peptide immunization with strong adjuvants to induce autoimmunity [24]. This might not be entirely reflective of spontaneous retinal autoimmunity given that the retina lacks lymphatic drainage [25–27], thus the immune cells involved with T cell priming and the effect of the local microenvironment on the priming might not be equivalent. Second is the growing realization that retinal MG are not a homogeneous population, but rather are likely to be composed of distinct subsets [28, 29]. This is evidenced by our findings of a retinal MG subset, identified as GFP^hi^ MG in the CD11c^DTR/GFP^ mouse that, as opposed to GFP^lo^ MG, expanded in numbers in response to injury and could act as conventional dendritic cells [5, 30, 31]. Thus, a change in the phenotype and number of retinal MG in response to a stimulus might not represent a change to all MG but rather a change in the balance and function between MG subsets.

In this study we sought to avoid the confounding factors associated with EAU induction by either adoptive transfer of in vitro activated T cells or immunization with adjuvants at remote sites in developing a better understanding of the origin and role of myeloid cells in retinal autoimmunity, particularly those that could be antigen presenting cells (APC), and the resulting T cell response. To do this, we employed R161H transgenic mice [32–34], which spontaneously develop autoimmunity in the retina and uveal tract we term spontaneous autoimmune uveoretinitis (SAU) to distinguish it from the uveoretinitis induced by immunization or adoptive transfer of T cells (EAU), in conjunction with CD11c^DTR/GFP^ mice. Using R161H^+/−^ × CD11c^DTR/GFP^ F_1_ mice and parabiosis, we report that SAU was delayed in the F_1_ mice relative to normal R161H mice, revealing a distinct prophase of the disease characterized by the expansion of retinal GFP^hi^ cells prior to observable autoimmunity, and that the induction of SAU strongly correlated with the recruitment of APC (GFP^hi^ cells) from the circulation into the retina.

## Methods

### Mice

R161H mice are a CD4^+^, alpha/beta T cell receptor transgenic mouse (B10.RIII background) specific for interphotoreceptor retinoid binding protein (IRBP) that begins to develop severe SAU by 3–4 weeks of age [32–34]. Breeders were provided by Dr. Rachel Caspi (National Eye Institute, National Institutes of Health). Wild-type B6/J mice and transgenic mice on the B6/J background were obtained from Jackson Laboratories. CD11c^DTR/GFP^ mice [2] express a chimeric membrane protein comprising green fluorescent protein (GFP) and diphtheria toxin receptor (DTR) under control of a transgenic CD11c promoter (Jackson Laboratories #004509). All R161H mice were bred and used as heterozygotes (R161H^+/−^) whether on the B10.RIII or B10.RIII × B6/J F_1_ background. Unless noted, all experiments were done with B10.RIII × B6/J F_1_ mice carrying the indicated transgene(s). The presence or absence of the transgene(s) was confirmed in all mice by PCR. B10.RIII-R161H-negative (R161H^−/−^) littermates were used as wild-type B10.RIII or as wild-type B10.RIII × B6/J F_1_ mice. All mice were *rd8* negative [35] with all R161H^−/−^ mice showing no evidence of retinal degeneration or inflammation. Mice were housed under cyclic light in specific pathogen-free conditions. Euthanasia was by CO_2_ inhalation.

### Parabiosis

All parabiotic pairs were made with B10.RIII × B6/J F_1_ mice to maintain MHC compatibility. Female mice were cohabitated for 7 days and then joined as described by Kamran et al. [36]. Post-surgical mice were maintained on daily 1-mL sub-cutaneous injections of lactated Ringer’s solution with 5% dextrose until normal feeding and hydration habits had resumed. For pairs joined 30 days or longer, blood from each parabiont was analyzed at 30 days post-surgery by flow cytometry for equivalent levels of circulating GFP^hi^ cells as confirmation of successful parabiosis. At the specified times the retinas of the parabiotic mice were examined by fundus imaging. At the termination of the experiment, the mice were euthanized, perfused, and separated for tissue harvest and analysis.

### Retinal fundus imaging

Mice were anesthetized with a combination of ketamine (80–100 mg/kg) and xylazine (8–10 mg/kg) given by intraperitoneal injection or by inhalation of 3% isoflurane to induce sedation then 1% isoflurane to maintain sedation. Pupils were dilated by a corneal application of 5 μL of a solution containing 2 parts 1.0% tropicamide and 1 part 0.5% proparacaine (as analgesic). Corneal hydration was maintained by liberal application of Systane or GenTeal. Retinal images were obtained using a Micron III retinal imaging system (Phoenix Technology Group). White light (brightfield) and fluorescence images were obtained. A 469/35-nm band-pass excitation filter in conjunction with a 525/50-nm band-pass emission filter was used for GFP detection. Clinical SAU was evaluated as described for EAU [37].

### Flow cytometry

Mice were euthanized, perfused, and the retinas removed as described [3, 5, 31, 38]. Retinas were individually dissociated at 37 °C for 30 min with a solution of 0.5 mg/mL Liberase/Blendzyme3 (Roche) and 0.05% DNase in calcium, magnesium-free Dulbecco’s phosphate buffered saline (DPBS) with gentle trituration. The retina samples were then incubated at 4 °C with the indicated fluorescent-labeled antibodies (BD Bioscience or eBioscience) for a minimum of 30 min, resuspended in DPBS with 2% fetal bovine serum (FBS) and analyzed using a BD Fortessa flow cytometer (BD Bioscience). An entire retina comprised a single sample, thus each sample represents the entire population of any specified immune cell(s) within that retina. Data analysis was done with FlowJo (Tree Star) software. Blood samples to confirm parabiosis were collected in DPBS with 2 units/mL heparin, stained with labeled anti-CD45 and anti-CD11b antibodies, lysed with 0.17 M NH_4_Cl (10 min at 37 °C), washed, resuspended in DPBS with 2% FBS, and analyzed for CD45^+^CD11b^+^GFP^+^ cells.

### Diphtheria toxin treatment

Depletion of GFP expressing cells in mice carrying the CD11c^DTR/GFP^ transgene was done with a single 100 μL (100 ng) intraperitoneal injection of diphtheria toxin (Sigma # D-7544).

### Histology

Eyes were preserved in Davidson’s fixative for a minimum of 24 h and then embedded in paraffin overnight. Transverse sections 6 μ thick were made through the central cornea to the optic nerve head and stained with hematoxylin and eosin (H&E). Histopathological SAU scores of 0 (no disease) to 5 (severe loss of photoreceptors plus damage to inner retinal layers) were based on changes to the retina as described for EAU [39].

### Statistical analysis

Statistical comparisons between animal groups were done as described in the “Results” section or figure legends.

## Results

### Appearance of spontaneous autoimmune uveoretinitis in R161H mice on the B10R.III × B6/J F_1_ background

**Fig. 1:**
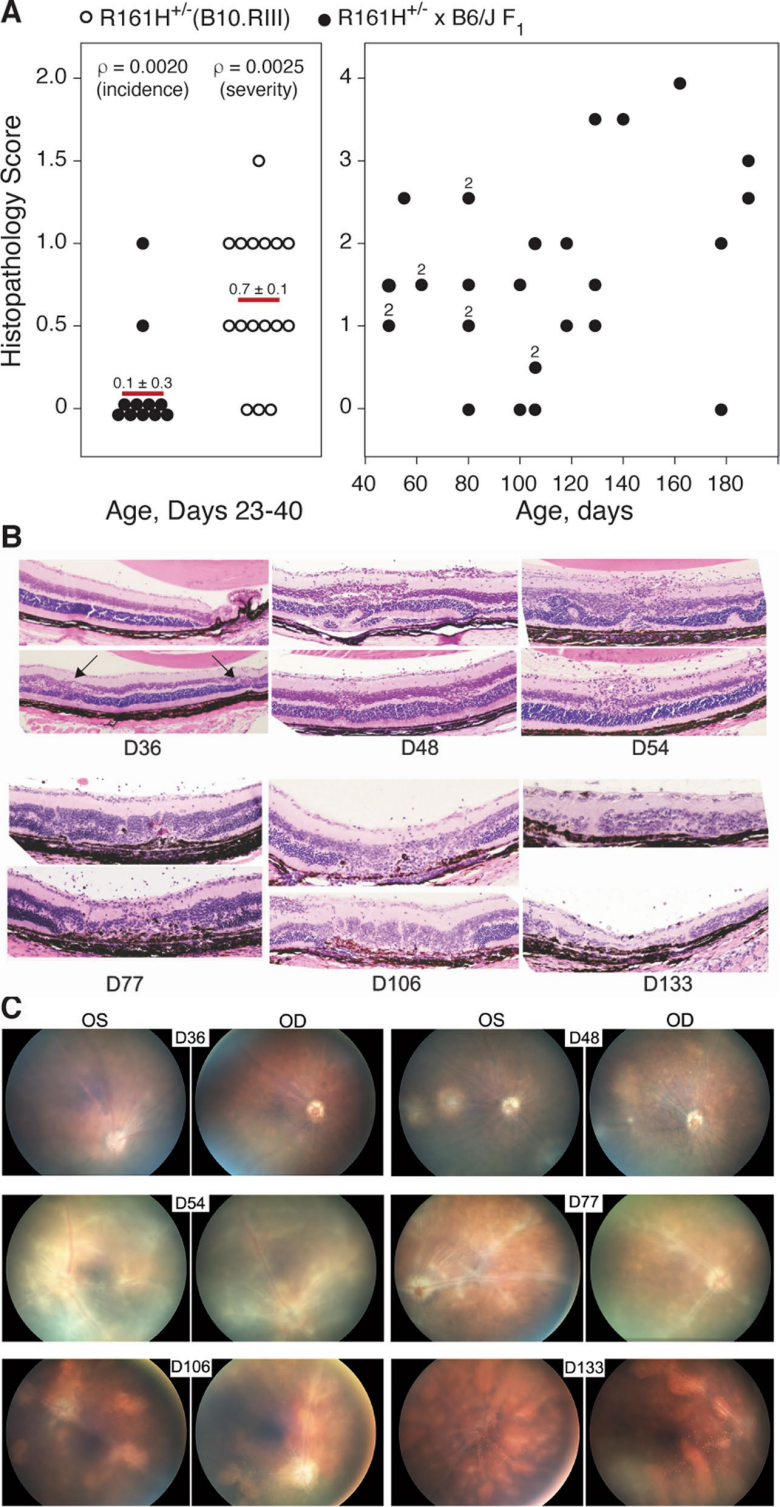
Progression of spontaneous autoimmune uveoretinitis (SAU). **A** Histopathological scoring of SAU in R161H^+/−^ (B10.RIII) mice (open circles, *n* = 16) and R161H^+/−^ × B6/J F_1_ mice (closed circles, *n* = 11) at days 23–40 of age (left panel, with mean ± SD indicated) and for R161H^+/−^ × B6/J F_1_ mice greater than 50 days of age (right panel, *n* = 29). Each data point represents one retina from one mouse. Number adjacent to data points represents number of retinas with that severity of SAU at that time point. For mice 23–40 days of age, statistical comparison between R161H^+/−^ (B10.RIII) and R161H^+/−^ × B6/J F_1_ mice was done by Fisher’s exact test for incidence and by *t* test for severity. **B** Representative histopathology in R161H^+/−^ × B6/J F_1_ mice over the course of disease. Right (OD) and left (OS) retinas from one mouse at indicated time point shown. Arrows on day 36 panel indicate initial minor pathology. **C** Brightfield fundoscopy of retinas from **B** showing clinical SAU

We adapted the R161H-B10.RIII SAU model [32–34] to the B10.RIII × B6/J F_1_ background to use currently available transgenic mice on the B6/J background that allow for the tracking of immune cells. To limit the rate of SAU induction and severity, and to compare the influence of the genetic background on pathology of SAU, all R161H^+^ mice were bred and analyzed as heterozygotes (R161H^+/−^). We compared the onset and severity of SAU in R161H^+/−^ mice (B10.RIII background) versus R161H^+/−^ × B6/J F_1_ mice between 23 and 40 days of age (Fig. 1A, left). Only 2/11 R161H^+/−^ × B6/J F_1_ mice exhibited minimal disease (SAU score mean ± SD = 0.1 ± 0.3). This is significantly different than the high incidence (80% incidence at 6 weeks of age) and severity reported elsewhere for R161H mice [32, 33, 40], as well as our own findings in this study in which 13 of 16 R161H^+/−^ (B10.RIII) mice developed SAU (SAU score mean ± SD = 0.7 ± 0.4) including 13/13 older than 28 days of age. Statistical comparison of R161H^+/−^ and R161H^+/−^ × B6/J F_1_ mice 40 days of age or less showed a significant difference in both incidence and severity (Fig. 1A, left). The earliest clinical or histological evidence of active inflammation in R161H^+/−^ × B6/J F_1_ retinas was observed at 36 days of age (Fig. 1B, C) with a gradual increase in retinal damage noted as R161H^+/−^ × B6/J F_1_ mice age (Fig. 1A–C). The delayed onset and reduced severity of SAU in R161H^+/−^ × B6/J F_1_ mice is advantageous in that it allows us to readily study immune cells in the course of a spontaneous autoimmune disease from pre-onset through resolution in a timely manner. The fact that most of the R161H^+/−^ × B6/J F_1_ mice eventually develop SAU (25/29, 86% for mice ≥ 50 days of age) confirms their utility for our studies (Fig. 1A, right).

### Analysis of T cell and myeloid cell infiltration in the course of spontaneous autoimmune uveoretinitis

**Fig. 2:**
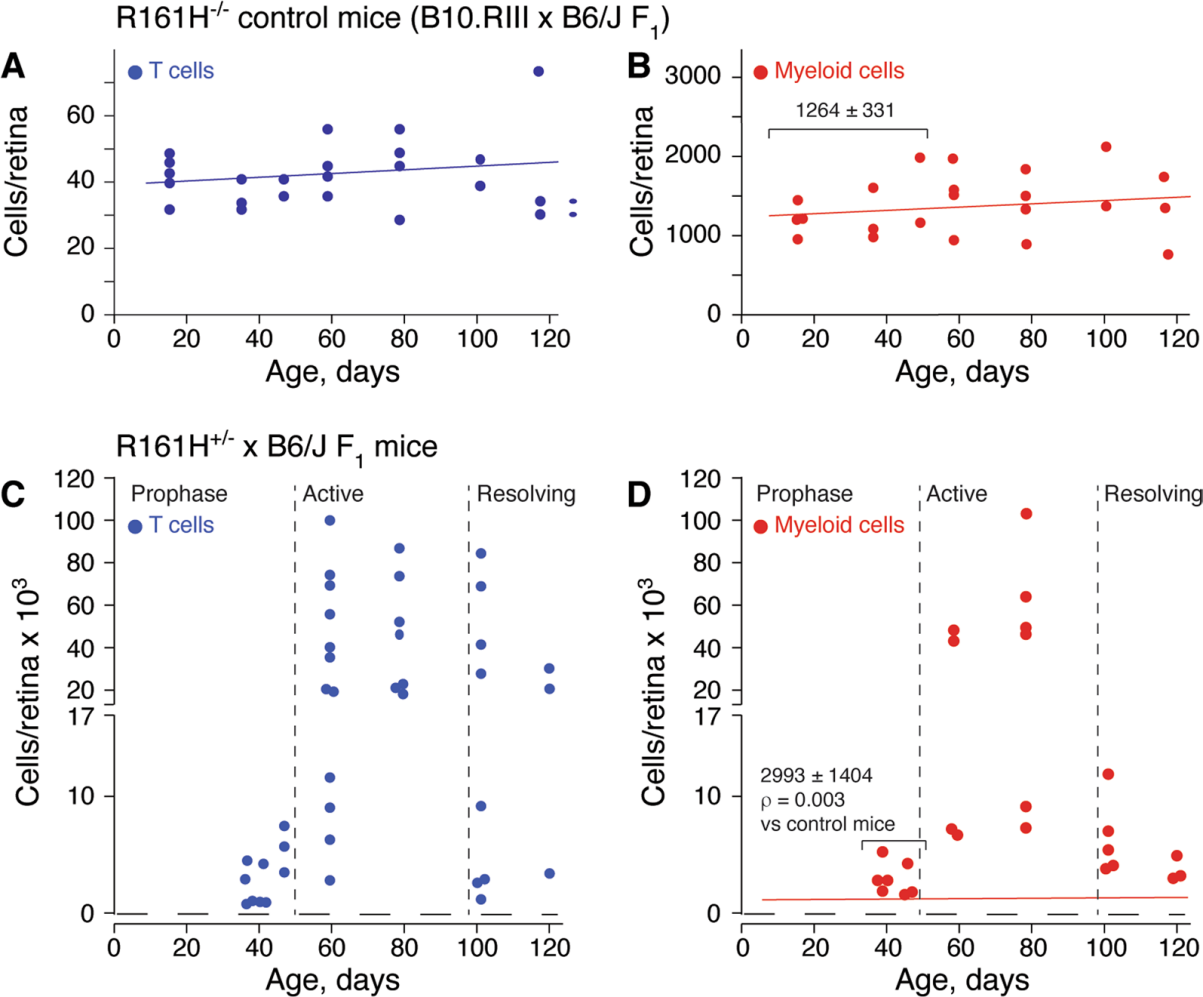
Quantification of retinal immune cells in SAU. **A**, **B** Retinal T cell and myeloid cell counts in control R161H^−/−^ × B6/J F_1_ (B10.RIII × B6/J F_1_) mice. Mean ± SD of retinal myeloid cells for mice ≤ 50 days of age indicated. Mean number of T cells and myeloid cells as function of age indicated by blue and red lines. **C**, **D** Kinetics of immune cell expansion in R161H^+/−^ × B6/J F_1_ mice. Prophase, active, and resolving phases of SAU are indicated. Mean ± SD of myeloid cells in prophase with statistical comparison (*t* test) to ≤ 50 day old control mice is indicated. For comparison, the mean number of myeloid cells in control B10.RIII × B6/J F_1_ retinas (from **B**) has been reproduced in **D** (red line). Myeloid cells were defined as CD45^med^CD11b^+^Ly6G^neg^ and T cells were defined as CD45^hi^CD90.2^+^Ly6G^neg^CD49^neg^. Cell numbers determined by flow cytometry

The conventional fundoscopy and histological analyses presented in Fig. 1 showed considerable variability in the timing of SAU onset and its rate of progression in R161H^+/−^ × B6/J F_1_ mice. However, quantitation of retinal myeloid cells and T cells from R161H^+/−^ × B6/J F_1_ mice ages 25 to 108 days revealed more predictable stages of SAU (Fig. 2). Expansion and contraction in the number of both classes of immune cells exhibited similar kinetics and could be divided into three phases. The first phase (prophase) was from approximately 25–45 days of age. While there is a significant increase in both T cells and myeloid cell numbers during prophase compared to the background levels in B10.RIII × B6/J F_1_ control mice (Fig. 2A, B versus C, D, *ρ* = 0.003 for myeloid cells) the eyes typically exhibit little or no histopathological damage to the retina (Fig. 1). The second phase (active phase, approximately 50–100 days of age) is marked by a very large and highly variable increase in retinal immune cells with widely variable clinical and histopathological damage. A third phase (resolving) occurs after approximately 100 days of age and represents the resolution of inflammation. Retinal immune cell numbers decrease with myeloid cells numbers approaching a level similar to that observed in SAU prophase. Damage to the retina was clearly evident by both clinical and histopathological examination.

### Characterization of immune cells in prophase of spontaneous autoimmune uveoretinitis

**Fig. 3:**
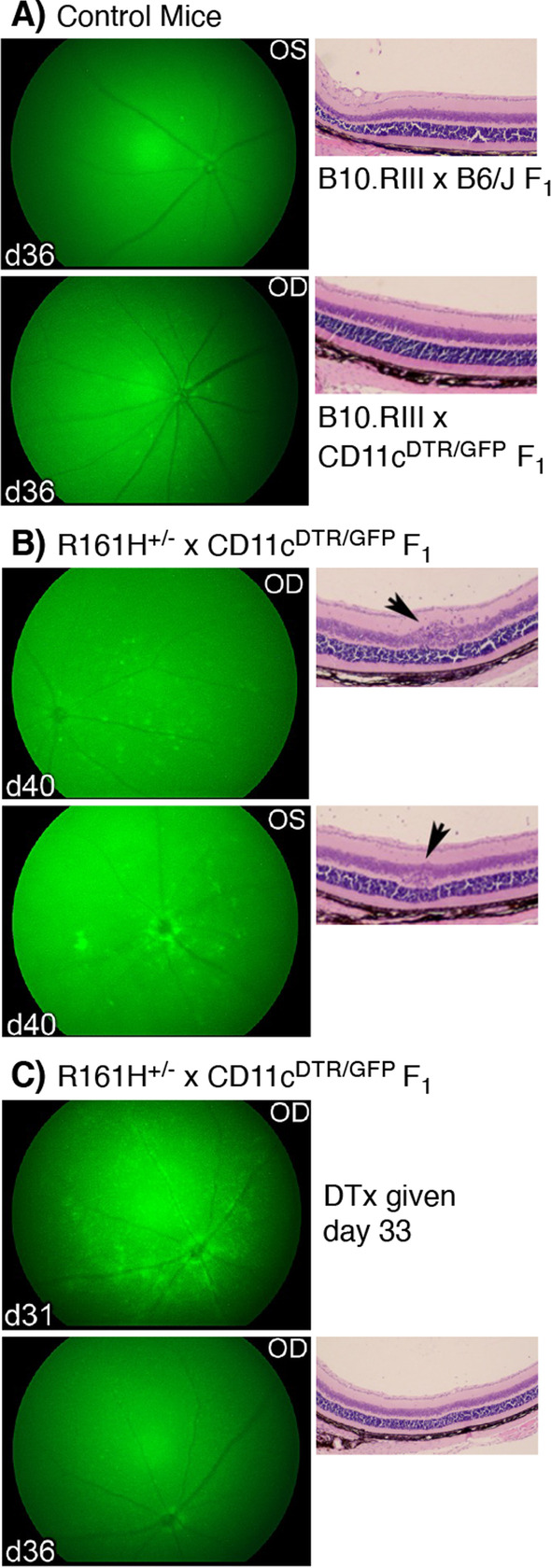
Fundoscopy and histopathological analysis of the prophase of SAU. **A** Retinas from B10.RIII × B6/J F_1_ and B10.RIII × CD11c^DTR/GFP^ F_1_ control mice analyzed at 36 days of age. **B** Fluorescence fundoscopy and histology of R161H^+/−^ × CD11c^DTR/GFP^ F_1_ retinas at 40 days of age showing a moderate and diffuse infiltration of GFP^hi^ cells and minimal histopathology (arrows). **C** Retinal images from an R161H^+/−^ × CD11c^DTR/GFP^ F_1_ mouse before (day 31) and after (day 36) diphtheria toxin (DTx) treatment on day 33 of age showing loss of GFP^hi^ cells due to DTx treatment and lack of histopathological damage. OS, OD = left and right retinas, respectively

As we have identified GFP^hi^ retinal MG in the CD11c^DTR/GFP^ reporter mouse as cells that have a dynamic population response to injury [5] and are essential for generating retinal T cell responses [31], we decided to further analyze the prophase immune cell response using R161H^+/−^ × CD11c^DTR/GFP^ F_1_ mice. The combination of fluorescence retinal fundus imaging combined with flow cytometry provided sensitive detection and quantification of the early expansion or infiltration of retinal immune cells. Analysis of control mice at 36 days of age confirmed both the expected lack of GFP expressing cells in the retina and lack of pathology in B10.RIII × B6/J F_1_ mice (Fig. 3A, top). B10.RIII × CD11c^DTR/GFP^ F_1_ mice examined at 36 days of age also lacked pathology but had the small number of widely scattered GFP^hi^ cells typical of these mice with quiescent retinas [4, 5] (Fig. 3A, bottom). Fundoscopic evidence of retinal GFP^hi^ cell expansion in R161H^+/−^ × CD11c^DTR/GFP^ F_1_ mice appeared as early as 29 days of age but was variable for onset and GFP^hi^ cells numbers during prophase (Fig. 3B, C). That the observed fluorescence was from cells expressing the DTR/GFP reporter transgene was confirmed by systemic diphtheria toxin treatment of the R161H^+/−^ × CD11c^DTR/GFP^ F_1_ mice which eliminated the fluorescence within 3 days (Fig. 3C). In addition to mice with diffuse retinal GFP^hi^ cells (Fig. 3B, C), we also observed prophase R161H^+/−^ × CD11c^DTR/GFP^ F_1_ mice with primarily perivascular GFP^hi^ cells, indicative of the earliest stages of retinal inflammation (data not shown). While the expansion of GFP^hi^ cells could be robust, histopathological evidence of T cell infiltration and retinal damage was usually minimal and not observed until later in prophase (Fig. 3B, arrows).

**Fig. 4:**
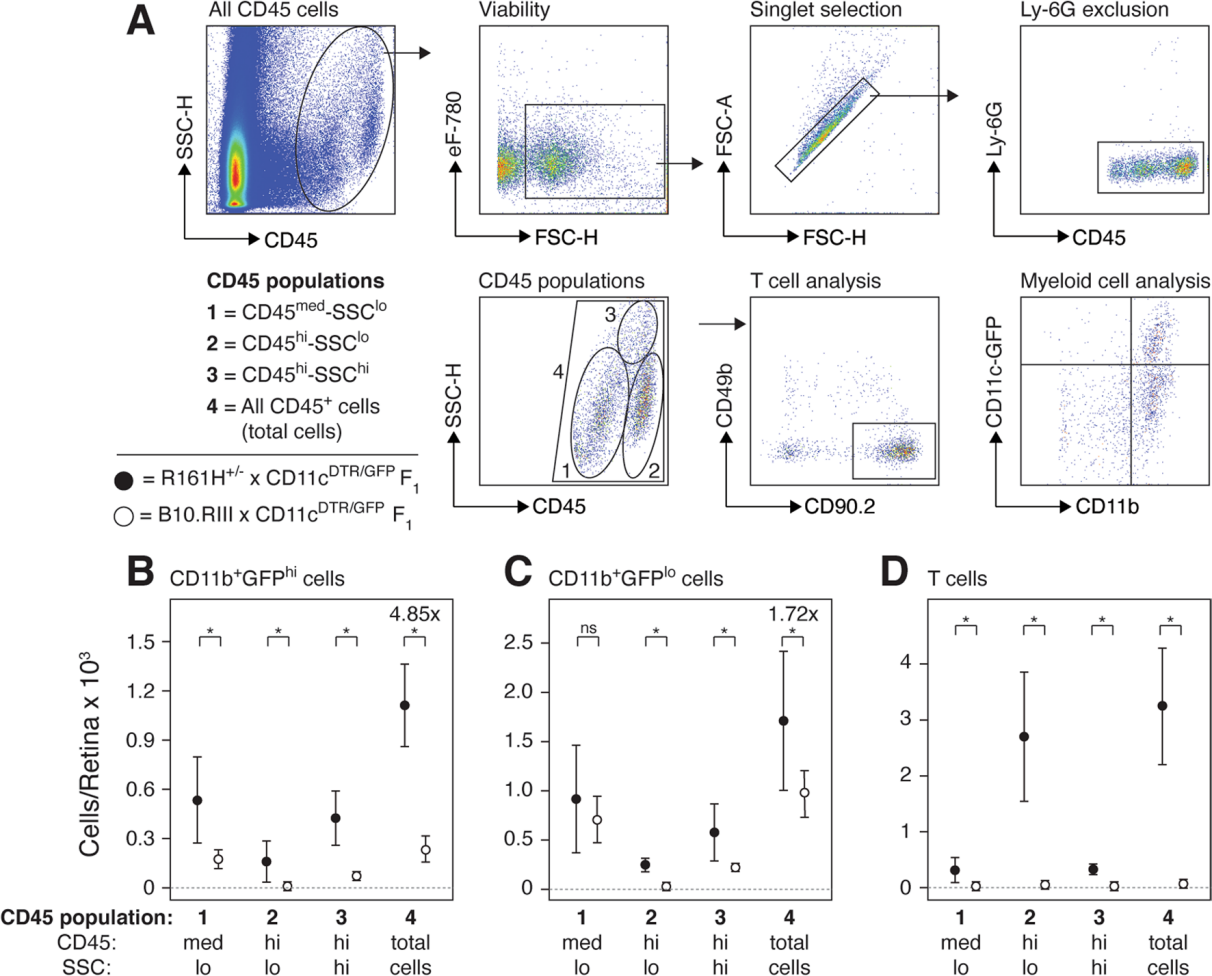
Analysis of myeloid and T cells in the retina during SAU prophase. **A** Flow cytometry gating strategy of a representative R161^+/−^ × CD11c^DTR/GFP^ F_1_ retina. CD45^+^Ly6G^−^ cells (top right panel) were divided into four populations based on their CD45 vs. SSC-H profile with each population then analyzed for T cells and myeloid cells. **B**–**D** Counts of retinal myeloid cells (CD11b^+^GFP^hi^ and CD11b^+^GFP^lo^ cells) and T cells from R161H^+/−^ × CD11c^DTR/GFP^ F_1_ (closed circles) and control B10.RIII × CD11c^DTR/GFP^ F_1_ mice (open circles). Numbers presented as mean ± SD, *n* = 6. Statistical comparison of R161H^+/−^ × CD11c^DTR/GFP^ F_1_ and control B10.RIII × CD11c^DTR/GFP^ F_1_ mice for each cell population is indicated (* = *ρ* < 0.01, ns = not significant by *t* test). Fold increase of total GFP^hi^ myeloid cells versus total GFP^lo^ myeloid cells in R161H^+/−^ × CD11c^DTR/GFP^ F_1_ and control B10.RIII × CD11c^DTR/GFP^ F_1_ mice is indicated (4.85× vs. 1.72×, *ρ* = 0.002 by *t* test)

Flow cytometry was used to quantify retinal myeloid and T cells in R161H^+/−^ × CD11c^DTR/GFP^ F_1_ mice at 36–40 days of age (Fig. 4). Although pathology was absent to minimal during prophase (Fig. 3B and C), flow cytometry confirmed significant increases in the number of GFP^lo^ and GFP^hi^ myeloid cells, as well as T cells, in R161H^+/−^ × CD11c^DTR/GFP^ F_1_ mice compared to B10.RIII × CD11c^DTR/GFP^ F_1_ control mice (R161H^−/−^ littermates). GFP^hi^ MG are a small portion of all MG in quiescent retinas, however they had a larger expansion in R161H-mediated SAU prophase (4.85× increase) than GFP^lo^ MG (1.72× increase) (Fig. 4B, C, total cell count). Elevated numbers of GFP^hi^ myeloid cells (Fig. 4B, left) were observed in the CD45^med^SSC^lo^ region indicating an expansion of resident GFP^hi^ MG or the conversion of resident GFP^lo^ MG to GFP^hi^ MG. In addition, there was also a significant increase in the number of GFP^hi^ myeloid cells in both the CD45^hi^SSC^lo^ and CD45^hi^SSC^hi^ regions suggesting their origin was circulating blood. Although modest, we also observed a statistically significant increase in GFP^lo^ myeloid cells that were either CD45^hi^SSC^lo^ or CD45^hi^SSC^hi^ in R161H^+/−^ × CD11c^DTR/GFP^ F_1_ (Fig. 4C, middle), again suggesting that R161H-mediated SAU can induce the recruitment of myeloid cells from the circulation into the retina. In contrast, there was no increase in the number of GFP^lo^ MG that were CD45^med^ (Fig. 4C, left). This population represents the non-APC resident MG of the retina, and its lack of expansion to autoimmune stimulation is consistent with the lack of expansion of GFP^lo^ MG we observed in our injury and neurodegeneration studies using CD11c^DTR/GFP^ mice [3–5]. R161H-mediated SAU prophase was also characterized by a large increase in T cells from about 40 that is typically observed in normal, quiescent retina (including B10.RIII × CD11c^DTR/GFP^ F_1_ retina) to over 3000 (Fig. 4D, see also Fig. 2C).

### Use of parabiosis to assess the role of circulating myeloid cells in the induction of spontaneous autoimmune uveoretinitis

**Fig. 5:**
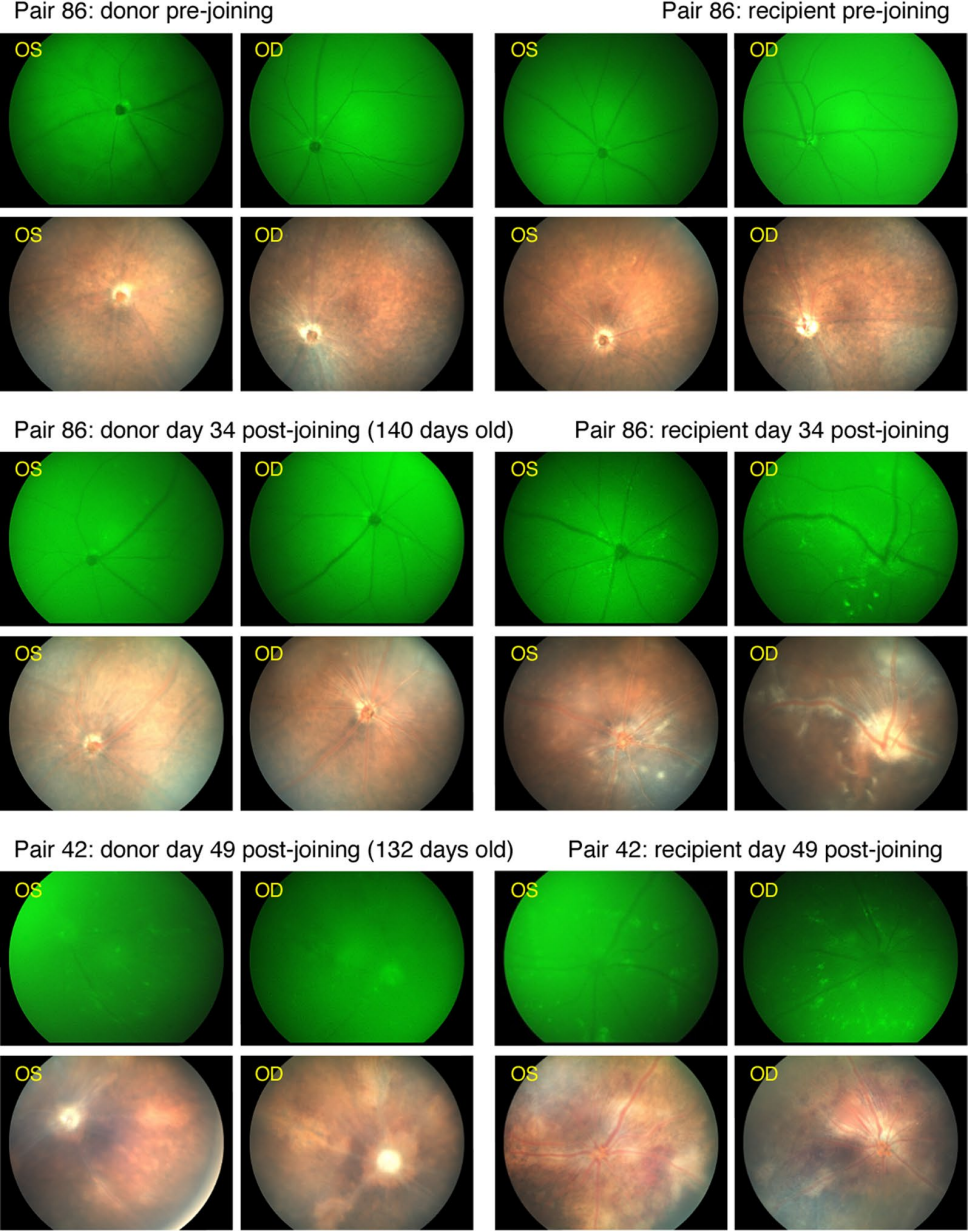
Representative fluorescence and brightfield fundoscopy of parabiotic retinas. Donor mice are R161H^+/−^ × CD11c^DTR/GFP^ F_1_ joined to B10.RIII × B6/J F_1_ (wild-type) recipients. Clinical SAU and GFP^hi^ cells are seen in recipient retinas. Pair 86 is the one pair (1/8) in which clinical SAU did not develop in the donor (donor age at joining 106 days) but was induced in the recipient. For pair 42 the donor was 83 days of age at joining and had active SAU. The duration of joining and total age of donor is indicated. OS, OD = left and right retinas, respectively

While it is known that severe autoimmunity can recruit CD11b^+^ cells into the retina ([22, 23] and above results), it has not been conclusively established whether resident or circulating myeloid cells are crucial for the induction of autoimmune disease in the retina. Since we have previously used parabiosis to demonstrate that the expansion of retinal myeloid cells in response to retinal degeneration and sterile mechanical injury models relies on resident progenitors within the retina or optic nerve [3, 4], we reasoned that parabiosis involving CD11c^DTR/GFP^ mice, which were developed to study the migration of dendritic cells (DC) into tissues [2, 41, 42], and the R161H mice would be useful in determining if the initiation of SAU was associated with the recruitment of circulating myeloid cells into the retina. Parabiotic pairs were made with R161H^+/−^ × CD11c^DTR/GFP^ F_1_ donor mice joined to B10.RIII × B6/J F_1_ (wild-type) recipients. This pairing allows for the transfer of autoreactive T cells and the transfer/tracking of circulating myeloid cells with dendritic cell-like antigen presentation capability (GFP^hi^ cells) into recipient retinas for the induction of SAU (Fig. 5).

As parabiosis is a novel approach for studying the transfer of retinal autoimmunity, we deemed it necessary to investigate parameters that could influence the success of disease transfer. We reasoned that the transfer of SAU might depend on several factors: age of donor mice, disease status of donor mice (no disease, active, or resolving), duration of joining, and age of recipient mice. Our results investigating these parameters and the association of disease induction with recipient retinas having recruited circulating (donor) APC are described below and summarized in Table 1. The results show that conditions associated with donor mice are most critical for the transfer of SAU.

**Table 1:** Parabiotic transfer of spontaneous autoimmune uveoretinitis (SAU)

Pairing condition	SAU incidence^a^	*ρ* value^b^
Donor < 67 days of age^c^	2/10	0.0180 vs. 71–165 days of age
Donor 71–165 days of age	10/14	
Donor > 175 days of age	0/5	0.0108 vs. 71–165 days of age
Donor: no or sub-clinical SAU^c^	1/8	0.0119 vs. active SAU
Donor: active SAU	10/14	
Donor: resolving SAU	1/7	0.0208 vs. active SAU
Paired 20–38 days	1/6	0.1824 vs. paired 43–153 days
Paired 43–57 days	6/11	0.1595 vs. paired 20–38 days
Paired 58–153 days	5/12	0.3058 vs. paired 20–38 days
Recipient: 44–63 days of age^c^	1/6	0.1824 vs. 64–177 days of age
Recipient: 64–85 days of age	3/10	0.5110 vs. 44–63 days of age
Recipient: 86–177 days of age	8/13	0.0913 vs. 44–63 days of age

Recipient retina outcomes	Occurrence^d^	*ρ* value^b^
GFP^hi^ cell positive	19/29	
SAU positive	12/29	
SAU positive/GFP^hi^ cell positive	12/19	0.0010 vs. GFP^hi^ cell neg/SAU pos
GFP^hi^ cell negative	10/29	
SAU positive/GFP^hi^ cell negative	0/10	

R161H^+/−^ × CD11c^DTR/GFP^ F_1_ (donor) joined with B10.RIII × B6/J F_1_ (wild-type recipient)

^a^Number of pairs with SAU positive recipients/total pairs for specified donor or recipient condition

^b^*ρ* value determined by Fisher’s exact test

^c^Age or SAU status at time of joining

^d^Number of recipient retinas with specified outcome/total pairs or pairs with specified GFP^hi^ cell outcome

#### Age of donor mice

Pairs with donors less than 67 days of age at time of joining transferred SAU at a low rate (SAU in 2/10 recipients) compared to pairs with donors between 71 and 165 days of age (SAU in 10/14 recipients). Some recipients from these groups had GFP^+^ cells in their retinas detectable by flow cytometry in the absence of any pathology visible by fundoscopy. Donors greater than 175 days of age failed to transfer SAU (SAU in 0/5 recipients), nor did we observe the transfer of GFP^+^ cells into the recipient retinas of pairs with these old donors.

#### Disease status of donor mice

As a corollary factor to donor age, we assessed the ability to transfer SAU from donor mice that had no signs of disease versus mice with active disease versus mice with clear evidence of resolving disease at the time of joining. Pairs with donor mice that were clinically negative for SAU (SAU in 1/8 recipients, see Fig. 5, pair 86 was the one pair with a clinically negative donor that transferred disease) or had obvious but resolved SAU (SAU in 1/7 recipients) transferred disease at a low rate compared to pairs in which the donor had or developed active SAU during the pairing (SAU in 10/14 recipients). Together this data shows the importance of active disease, a state with the highest number of circulating myeloid cells with APC capability and effector T cells in donor mice, in the transfer of SAU to recipient mice. Since it has been reported that Tregs are associated with the resolution of disease in EAU models [31, 43–45], it is possible that T cells in older donors or those with resolved SAU could be skewed towards a Treg phenotype potentially limiting their ability to transfer SAU.

#### Duration of joining

We assessed whether development of SAU in recipient mice was dependent on time of exposure to circulating R161H^+/−^ T cells. Pairs joined for 20–38 days transferred disease at a low rate (SAU in 1/6 recipients) while those paired for 43–57 days transferred disease at a higher rate (SAU in 6/11 recipients). The longest duration pairings of 58–153 days also transferred SAU at a higher rate (SAU in 5/12 recipients), albeit slightly reduced compared to those paired for 43–57 days. These longest duration pairings often resulted in resolved SAU in the donor mouse, a factor that could limit the transfer of SAU. Although the difference in the rate of SAU development in the longer-term pairings was not statistically significant compared to the shorter-term pairings, the enhanced incidence of disease in recipient mice after day 43 reflects the increased incidence of SAU in unpaired R161H^+/−^ × B6/J F_1_ mice after 40 days of age (Fig. 1).

#### Age of recipient

We also analyzed the transfer of SAU as a function of the recipient mouse age at time of joining. Wild-type F_1_ recipient mice 44–63 days of age were minimally susceptible for developing SAU (SAU in 1/6 recipients). Recipients older than 64 days of age had an increased, but not statistically significant, incidence of SAU regardless of grouping (SAU in 11/23 total recipients), nor was there a significant difference in the rate of SAU in mice between 64–85 days of age versus 86–177 days of age (SAU in 3/10 vs. 8/13 recipients, *ρ* = 0.1402).

#### Correlation of recipient SAU and recruited circulating APC

Fundoscopic analysis and/or flow cytometry analysis of the 29 pairings between R161H^+/−^ × CD11c^DTR/GFP^ F_1_ mice and B10.RIII × B6/J F_1_ wild-type recipients resulted in detectable GFP^hi^ cells in the retinas of 19 recipients (Table 1, bottom). Of the GFP^hi^ cell-positive recipients, 12 showed SAU by clinical and/or histopathological exam which indicated a correlation between recruitment of GFP^hi^ cells to recipient retinas and the induction of SAU. As the donor GFP^hi^ cells simply represent the equivalent GFP^neg^ antigen presenting cells also circulating in the recipient mice, the data in a more general sense, suggest that recruitment to the retina of circulating antigen presenting cells is important for the induction of autoimmune uveoretinitis. We also observed GFP^hi^ cells in recipient retinas (7 of 19 GFP^hi^ cell-positive recipients, Table 1) without evidence of SAU. This observation is likely analogous to the situation observed in the prophase portion of SAU in unpaired R161H^+/−^ F_1_ mice (Figs. 2 and 3) where SAU is often not clinically detectable. Conversely, 10 of the 29 wild-type recipients were negative for GFP^hi^ cells and all of them were negative for SAU. The incidence of SAU in recipient retinas with GFP^hi^ cells was highly significant compared to retinas without GFP^hi^ cells (*ρ* = 0.0010, Table 1, bottom). The inability to induce SAU in parabiotic recipients without presence of retinal GFP^hi^ cells further demonstrates that induction of retinal autoimmunity likely requires recruitment of antigen presenting cells from the circulation.

As a control for the recruitment of circulating GFP^hi^ cells to the retina in the absence of a stimulatory event, B10.RIII × CD11c^DTR/GFP^ F_1_ mice (donor) were parabiosed with B10.RIII × B6/J F_1_ mice (wild-type recipient). The small number of GFP^hi^ cells in normal, quiescent CD11c^DTR/GFP^ retinas (typically 100–200) are difficult to visualize by fundoscopy but can be readily detected by flow cytometry [5, 6]. Donor and recipient retinas from these control parabiotic mice were analyzed by flow cytometry for GFP^hi^ cells after being paired for at least 50 days. GFP^hi^ cells were found in donor but not recipient retinas confirming that the appearance of GFP^hi^ cells in wild-type recipients paired with R161H^+/−^ × CD11c^DTR/GFP^ F_1_ donors was associated with the induction of SAU (data not shown). In a previous study, we parabiosed mice with β-actin promoted GFP expression (ACTβ^eGFP^) to normal B6/J mice up to 6 months and found that the B6/J recipient retinas had ≤ 10 GFP^+^ cells and that number did not increase even after stimulation by optic nerve crush [4]. Since the number of circulating hematopoietic cells expressing GFP is much less in CD11c^DTR/GFP^ mice than ACTβ^eGFP^ mice it was not surprising to find no GFP^hi^ cells in the retinas of the B10.RIII × B6/J F_1_ recipients when paired with B10.RIII × CD11c^DTR/GFP^ F_1_ mice.

**Fig. 6:**
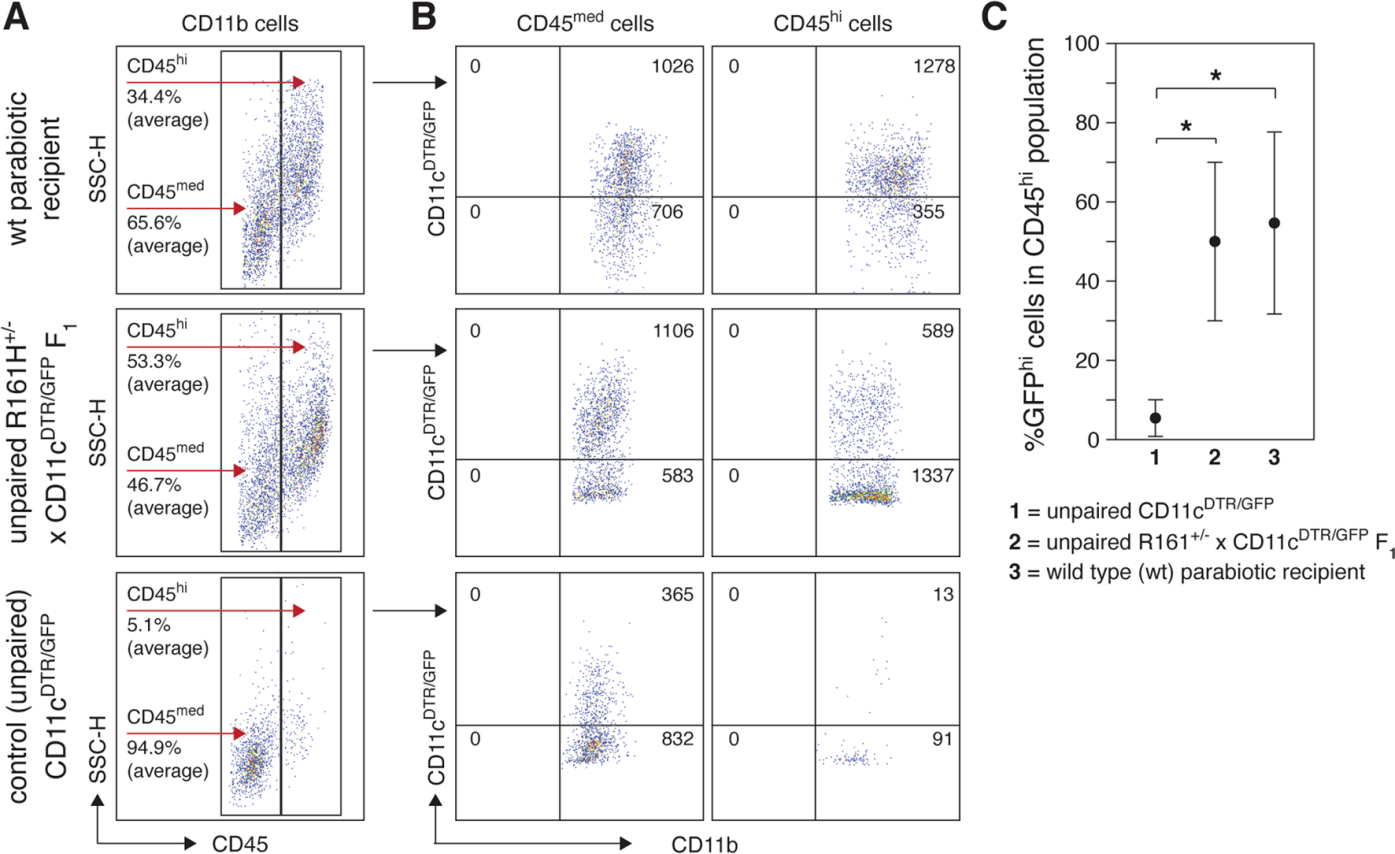
Flow cytometry analysis of retinal CD11b cells from parabiotic recipient mice. Donor mice are R161H^+/−^ × CD11c^DTR/GFP^ F_1_ joined to B10.RIII × B6/J F_1_ (wild type) recipients. **A** CD11b^+^ cells from representative parabiotic wild-type recipient (top), unpaired prophase R161H^+/−^ × CD11c^DTR/GFP^ F_1_ (middle), and control CD11c^DTR/GFP^ (bottom) retinas. Retinal CD45^+^ cells were selected as described in Fig. 4 with CD45^+^Ly6G^neg^ cells further gated to select for CD11b cells (CD11b^+^CD49b^neg^CD3^neg^CD4^neg^). CD11b^+^ cells were then analyzed for CD45 expression levels versus SSC-H with CD45^med^ and CD45^hi^ cell populations and overall average percentage indicated. **B** Representative analysis of retinal CD11b^+^CD45^med^ and CD11b^+^CD45^hi^ cells for GFP expression with cell numbers indicated. **C** Graphical comparison of GFP expression in CD11b^+^CD45^hi^ cells from unpaired (control) CD11c^DTR/GFP^ mice (*n* = 6), unpaired (control) R161^+/−^ × CD11c^DTR/GFP^ F_1_ mice (*n* = 4), and parabiotic wild-type recipient mice (*n* = 4). Data shown as mean ± SD, * = *ρ* < 0.05 by *t* test

We and others have demonstrated that circulating monocytes entering CNS tissue in general [46, 47], and the retina in particular [6, 48], display a high level of CD45 expression compared to resident MG but down-regulate their CD45 levels as they reside in the retina to that resembling endogenous MG [6, 46]. Thus, we reasoned that the appearance of CD45^hi^ myeloid cells, particularly CD11b^+^GFP^hi^ cells, in wild-type parabiotic recipient retinas was evidence that recruitment of APC from the circulation was a prerequisite for the induction of SAU. Myeloid cells from the retinas of wild-type recipients parabiosed for 43–83 days that showed some evidence of SAU induction by fundoscopy (presence of GFP^hi^ cells or retinal inflammation) were analyzed by flow cytometry and compared to myeloid cells from the retinas of unpaired R161H^+/−^ × CD11c^DTR/GFP^ F_1_ mice in prophase (30–50 days of age) (Fig. 6). This comparison was chosen as it makes the exposure time to the autoreactive R161H T cells similar. Initial analysis of all retinal CD11b^+^ cells showed that an average of 34.4% were CD45^hi^ in the wild-type parabiotic recipient mice compared to 53.3% in unpaired R161H^+/−^ × CD11c^DTR/GFP^ F_1_ mice in prophase and 5.1% in CD11c^DTR/GFP^ control mice (Fig. 6A, *ρ* < 0.001 for parabiotic recipient and unpaired R161H^+/−^ × CD11c^DTR/GFP^ F_1_ versus control CD11c^DTR/GFP^, *ρ* = not significant for parabiotic recipient versus unpaired R161H^+/−^ × CD11c^DTR/GFP^ F_1,_ by *t* test). The CD11b^+^CD45^med^ and CD11b^+^CD45^hi^ populations from the parabiotic recipients, unpaired R161H^+/−^ × CD11c^DTR/GFP^ F_1_ mice, and CD11c^DTR/GFP^ control mice were then analyzed for GFP expression (Fig. 6B). In the representative retinas shown in Fig. 6B, 55.5% (1278/2304) of the putative CD11b^+^GFP^hi^ APC from the parabiotic recipient were CD45^hi^ and from the unpaired R161H^+/−^ × CD11c^DTR/GFP^ F_1_ it was 34.7% (589/1695) versus 3.4% (13/378) from the unpaired CD11c^DTR/GFP^ control mice. Overall, we found that 56.9% of the CD11b^+^GFP^hi^ cells from the parabiotic recipient retinas were CD45^hi^ while 55.0% from the unpaired R161H^+/−^ × CD11c^DTR/GFP^ F_1_ were CD45^hi^ compared to 5.9% from unpaired CD11c^DTR/GFP^ control retinas (Fig. 6C). As the parabiotic recipients are initially devoid of GFP^+^ cells it demonstrates that the 43.1% of the retinal CD11b^+^GFP^+^ cells that are CD45^med^ originated in the circulation and have resided in the retina long enough to down-regulate CD45. The overall similarity of CD11b^+^GFP^hi^ cells that are CD45^hi^ between unpaired R161^+/−^ × CD11c^DTR/GFP^ F_1_ mice in SAU prophase and parabiotic recipients validates our parabiotic model for studying the origin of APC associated with retinal autoimmunity. The wide range of all CD11b^+^ cells and the CD11b^+^GFP^hi^ subset that are CD45^hi^ for individual mice within the parabiotic recipients and unpaired R161^+/−^ × CD11c^DTR/GFP^ F_1_ groups can be attributed to the highly variable progression of SAU and the down-regulation of CD45 expression that occurs as newly recruited myeloid cells take up residence in the retina [6].

In summary, (1) the presence of GFP^hi^ cells, regardless of their CD45 status in wild-type parabiotic recipient retinas, requires that they entered from the circulation; (2) there is a strong correlation between GFP^hi^ cells in recipient retinas and the development of SAU, and (3) a significant percentage of the CD11b^+^GFP^hi^ APC in recipient retinas are CD45^hi^, characteristic of circulating myeloid cells recently recruited to the retina. These experiments provide multiple lines of evidence that induction of SAU is highly associated with the recruitment of circulating APC to the retina.

## Discussion

It has been well established that T cell-mediated autoimmunity in non-immune privileged tissue requires circulating APC. However, in immune privileged sites, particularly CNS tissue, demonstrating a requirement of circulating APC for induction of autoimmunity has been a significant challenge [49–51]. CNS tissues exhibit a wide range of responses to injuries and inflammatory events that can involve the expansion of resident myeloid cells as well as the recruitment of significant numbers of lymphoid and myeloid cells from the circulation. In certain models of neuroinflammation such as experimental autoimmune encephalomyelitis (EAE), a murine model of human multiple sclerosis, circulating APC are thought to be necessary for the induction and progression of the disease [52–55]. Although the retina is a part of the CNS, there is growing evidence that it is unique in its ability to locally control and regulate immune responses compared to other CNS tissue [6, 56, 57]. The retina lacks meninges, dura mater, subarachnoid space, and choroid plexus, all of which are known to have a substantial population of circulation-derived macrophages [1], thus making it a truer representation of neural parenchyma. In retinal autoimmune conditions, such as uveoretinitis in humans and EAU in rodents, the blood retinal barrier, the presence of resident macrophages and MG, as well as the difficulty in discriminating between circulating and resident myeloid cells has left in question the origin and nature of the APC that are responsible for initiating immunopathology compared with cells whose presence is simply a consequence of tissue damage. The retina is particularly well suited to study the issue of expansion and/or recruitment of immune cells to CNS tissue since, in its quiescent state, it has a small and easily quantifiable number of immune cells thus allowing changes in response to immunological challenges to be readily detected. Further, the immune cell response within a retina can be visualized and analyzed over the course of the event, especially in mice where the immune cell(s) of interest express a fluorescent label.

In studying retinal immune responses, we have employed a variety of strategies looking for evidence that the presence of specific recruited immune cells versus the expansion of resident immune cells reflects the nature of the challenge and the inherent goal of preserving vision. We have demonstrated there is a subset of retinal MG that dynamically responds to non-autoimmune inflammatory events [3–5]. Further, we and others have shown that, in the absence of severe damage to normal physiological barriers induced by radiation or traumatic injury, retinal MG subsets are replenished by and/or expand from resident myeloid cells within the retina or adjacent CNS tissue [4, 6, 12–15]. Although these and other similar studies represent a significant body of research into the response of retinal innate immune cells, they did not elucidate the nature and origin of APC associated with conditions leading to the onset of retinal autoimmunity.

Several early studies led us to propose that APC recruited from the circulation could be critical for EAU pathogenesis. First was our observation that CD45^+^ cells from quiescent murine retina were poor in their ability to present antigen (act as conventional DC) and stimulate an effector response from naive T cells compared to splenic and even brain-derived CD45^+^ cells [56]. Second was our study using radiation bone marrow chimeras between EAU susceptible (Lewis) and non-susceptible (Brown Norway and Buffalo) rat strains showing that EAU induction, following adoptive transfer of activated retinal S-Ag specific T cells, depended on the chimeric rats having circulating monocytes derived from engrafted Lewis bone marrow or splenocytes [58]. While these results suggested that circulating APC were crucial for EAU induction, we could not discount the possibility that proinflammatory cytokines produced by the activated T cells and/or their intrusion into the retina led to the activation of retinal cells capable of acting as APC. This would remain an issue in studies attempting to determine the origin of APC associated with EAU induction as long as the disease was induced by adoptive transfer of activated T cells or immunization at distal sites.

Our experience with a trackable myeloid cell capable of expansion within or recruitment to the retina that could act as a conventional DC (retinal GFP^hi^ cells in CD11c^DTR/GFP^ mice), along with the development of the R161H mouse model of SAU [32–34], allowed us to reexamine whether the induction of retinal autoimmunity was associated with the recruitment of circulating APC to the retina. For several reasons, we found it both useful and necessary to analyze F_1_ mice resulting from crossing R161H^+/−^ (B10.RIII) mice to B6/J mice, a strain permissive but less susceptible for EAU [24, 59]. First, as most transgenic mice with immune cell markers are on the B6 background, an F_1_ was required for use of our CD11c^DTR/GFP^ mice. Second, as SAU occurs at a young age and progresses rapidly to severe retinal destruction and resolution in R161H^+/−^ (B10.RIII) mice, we reasoned that R161H heterozygous mice on a partial B6/J background could have delayed onset and slower disease progression, providing the opportunity to study the early events and immunopathogenesis of retinal immune cell responses using parabiosis. Finally, F_1_ mice were necessary to ensure MHC compatibility for parabiosis. Although there was a risk of R161H^+/−^ × B6/J F_1_ mice having a low incidence of SAU, our observation that most R161H^+/−^ × B6/J F_1_ eventually developed retinal autoimmunity that was delayed and reduced in severity compared to R161H^+/−^ (B10.RIII) mice made the F_1_ model useful in the present studies. Further, the F_1_ model provides a platform unencumbered by the limitations of distal immunization and adoptive T cell transfer for further studies on the origin, role, and response of immune cells in retinal autoimmunity.

Using R161H^+/−^ × B6/J F_1_ mice, we characterized the development of SAU as having three distinct phases based on immune cell numbers within the retina. Although the prophase, active disease, resolution pattern is likely typical of tissue-specific autoimmune responses, the slow progression of SAU in F_1_ mice allowed us to closely examine the nature of retinal myeloid cells early in the disease process. In analyzing prophase retinas from R161H^+/−^ × CD11c^DTR/GFP^ F_1_ mice, we observed a preferential increase in GFP^hi^ myeloid cell numbers compared to GFP^lo^ myeloid cells suggesting an important role for the GFP^hi^ MG subset either in the induction or progression of SAU. We also observed that a significant percentage of the retinal GFP^hi^ cells were also CD45^hi^ indicating they were derived from the circulation. In addition, the more modest increase in GFP^lo^ myeloid cell numbers from prophase retinas was also characterized by those cells also being primarily CD45^hi^, again suggesting a recruitment of myeloid cells from the circulation. While these results provide circumstantial evidence that circulating APC are likely necessary for induction of SAU or EAU, it could not determine whether circulating GFP^hi^ cells (or precursors thereof) or GFP^lo^ cells were the crucial APC, nor could it eliminate the possibility the signals provided by infiltrating myeloid and lymphoid cells up-regulated CD45 and GFP expression in resident myeloid cells of the retina.

Parabiosis has become a useful tool for studying the origin and contributions of myeloid cells in CNS and peripheral lymphoid tissues under normal and pathological conditions [1, 9, 55, 60, 61]. For our purposes we deemed it necessary that both the activated, autoreactive T cells (R161H) and the labeled, putative circulating APC (GFP^hi^ cells) be in one mouse (the donor) while the recipient mouse remain wild-type. While our system is not designed to answer every question about what is needed for autoimmunity it does answer a particular question—is the recruitment of cells from the circulation known to be APC (GFP^hi^ CD11c^+^ cells) associated with retinal autoimmunity. As such, it remains a formal possibility that other circulating hematopoietic cells could also be involved with the induction of SAU. Investigating this would require the use of CD45 congenic markers in combination with our system. However, we believe the limited variability of our system is actually advantageous in that it investigates a specific function of a defined set of cells. It must be noted that our system does not provide a gain of function for the recipient mice but rather simply provides a reporter for circulating CD11c^+^ APC that would be present in any mouse.

In analyzing our parabiotic mice, we found that donor age at joining, duration of joining, and the activity of SAU in the donor mouse at time of joining were important factors in the appearance of uveoretinitis in the recipient. Parabiotic mice joined for less than 38 days exhibit a low incidence of disease in recipient mice. Although we did not analyze for chimerism of the circulation in parabionts joined less than 30 days, we believe the low incidence of SAU in these recipients was due to factors associated with its normal course of pathology rather than poor chimerism as full chimerism of circulating hematopoietic cells occurs by 7 to 14 days in parabiotic mice [36, 62]. Active SAU in the R161H donor mouse led to efficient transfer of the disease to recipient mice. Conversely, R161H donor mice with either sub-clinical or resolving SAU poorly transferred disease to recipient mice. The generation of regulatory T cells (Tregs) is crucial for the control and resolution of EAU [31, 43–45] and we have observed increased regulatory/effector T cell ratios in the retinas of R161H^+/−^ × B6/J F_1_ mice with resolving SAU (unpublished observation). Thus, it is possible that the poor transfer of SAU associated with convalescent donors is due to increased numbers and activity of Tregs from the donor mouse.

However, the most important observation from our experiments with parabiotic mice was that development of SAU in recipient mice was highly associated with the appearance of GFP^hi^ APC in recipient retinas. We found GFP^hi^ cells in the retinas of 19 of 29 recipients with 12 of those developing SAU. Further, we observed that the percentage of recipient retina GFP^hi^ cells that were CD45^hi^ was equivalent to unpaired R161H^+/−^ × CD11c^DTR/GFP^ F_1_ mice at prophase of SAU. This indicated that there was a faithful replication of the disease in the parabiotic recipients and that the GFP^hi^ cells originated in the circulation. As the onset of clinically or histologically observable SAU is variable, the lack of SAU in any recipient positive for retinal GFP^hi^ cells was likely due to insufficient time between GFP^hi^ cells entering recipient retinas and the time of final analysis. However, we could not discount other factors including Treg activity. Conversely, in recipients that lacked retinal GFP^hi^ cells we did not observe SAU. Since donor and recipient mice were joined long enough to establish chimerism, the circulation within every parabiont contains labeled APC (donor GFP^hi^ cells), the equivalent but unlabeled APC from the recipient, and the R161H autoreactive T cells. As such, and regardless of the disease state of the donor, wild-type recipient parabionts are prime for developing SAU. The successful induction of retinal autoimmunity likely depends on a cascade of signals and events. Although the signal(s) that begin the process of recruiting circulating APC to the retina remain unresolved, our data is clear that a migration of APC from the circulation into the retina appears to be required for the induction of SAU or EAU. Just as important, if the resident retinal microglial APC could support an effector T cell response, we would expect to see pathology in our wild-type parabiotic recipients even in the absence of donor GFP^hi^ cells; this was not observed.

In summary, our results highlight a unique facet of retinal immunology in that circulating, but not resident, APC are likely required for retinal autoimmune pathology. This contrasts with our findings on optic nerve crush injury and cone degeneration associated with RPE65 deficiency showing that recruitment of circulating myeloid cells to the retina is not part of the response to those conditions [3, 4]. A key difference between these retinal insults is the involvement of T cells. Retinal T cells remained at background levels in our injury and non-inflammatory degeneration models while there was a large influx of T cells into the retinas of R161H^+/−^ × B6/J F_1_ mice even with only minimal levels of clinical or histopathological inflammation. As it has been demonstrated that activated, retinal antigen-specific T cells readily cross the blood retina barrier [40, 63, 64] we postulate that their arrival in the retina signals for recruitment of circulating myeloid cells capable of acting as APC. In the case of R161H mice the initial T cell activation occurs by recognition of cross-reactive epitopes present on commensal microbiota [40]. We have studied other retinal-antigen specific T cell receptor transgenic systems and found other T cell stimulating factors such as lymphopenia and Treg depletion were required for T cells activation and subsequent autoimmunity [31, 65]. Although we have demonstrated there is a subset of retinal MG that can act as conventional DC, it is highly dependent on there being a stimulatory event such as an injury [5, 30]. In the absence of such an event, infiltrating T cells encounter myeloid cells in an environment that is not conducive for the initiation or progression of autoimmunity. Thus, the induction of retinal autoimmunity likely requires the recruitment of circulating APC which have not been altered by the immunosuppressive environment of the quiescent retina.

Although principally explored in neural tissue, a dynamic between embryonically derived tissue resident macrophages and circulating myeloid cells in homeostasis and disease is emerging in other tissues [51, 66]. Neuroinflammation and autoimmunity in general are often treated with broad immunosuppressive therapies such as corticosteroids or modulators of T cell activation and effector functions that can interfere with the protective and homeostatic functions of the immune system. It has been proposed that therapies specifically targeting antigen presentation could be effective against autoimmune disorders while limiting the adverse effects associated with broader systemic approaches (67). Our work suggests that limiting recruitment of circulating APC into tissues could be an effective strategy in limiting autoimmune conditions particularly those involving CNS tissue.

## Conclusions

As with any T cell-mediated autoimmune condition, cells of the innate immune system have long been recognized as crucial to the induction and resolution of retinal autoimmunity. However, difficulties in differentiating resident microglia from monocytic macrophages that infiltrated CNS tissue from the circulation have made determining whether it is resident or recruited APC that are responsible for inducing the autoimmunity a challenge. Resolution of this issue has also been hampered by limitations of the model systems used to study it. Our work combines a new model of spontaneous retinal autoimmunity, a method for tracking APC capable of residing and/or infiltrating the retina that can induce an effector T cell response, and parabiosis to show that the induction of retinal autoimmunity is clearly associated with the recruitment of circulating APC into the retina as opposed to the response associated with non-inflammatory injury or neural degeneration which exclusively utilizes resident MG. Although these conditions can induce a subset of retinal MG to act as conventional DC, our finding that it takes newly recruited APC to support an autoimmune response within the retina confirms the immunosuppressive bias of the retinal microenvironment and its resident myeloid cells.

## Data Availability

Data and all materials except for R161H mice are available on request from the corresponding author. R161H mice are not commercially available. For availability and use of R161H mice contact their developer Dr. Rachel R. Caspi, National Eye Institute, caspir@nei.nih.gov.

## Abbreviations

APC: Antigen presenting cells
CNS: Central nervous system
DC: Dendritic cells
DTR: Diphtheria toxin receptor
EAE: Experimental autoimmune encephalomyelitis
EAU: Experimental autoimmune uveoretinitis
GFP: Green fluorescent protein
MG: Microglia
SAU: Spontaneous autoimmune uveoretinitis
Treg: Regulatory T cells
